# Optical Coherence Tomography Imaging: Advances in Ophthalmology

**DOI:** 10.3390/jcm11102858

**Published:** 2022-05-18

**Authors:** Sumit Randhir Singh, Jay Chhablani

**Affiliations:** 1 Nilima Sinha Medical College & Hospital, Rampur 852122, India; sumit.jipmer@gmail.com; 2 UPMC Eye Center, University of Pittsburgh, Pittsburgh, PA 15213, USA

Since its advent in 1991, optical coherence tomography (OCT) has become the most commonly used imaging modality in vitreo-retina practice [1]. OCT, a non-invasive imaging modality, has a fast acquisition time, usually within seconds, and provides in vivo, high resolution, three-dimensional (3-D) imaging of the retina and choroid, akin to the histologic section [2]. These inherent advantages have enabled OCT installation in eye clinics throughout the world, thereby providing invaluable insights about the chorioretinal architecture in diverse ocular diseases.

Based on the principle of low coherence interferometry, OCT uses an infrared light wavelength ranging from 840 nm to 1050 nm [3]. Several technical modifications from the earlier time domain OCT (TD-OCT) to recent upgrades, including spectral domain (SD-OCT) and swept source OCT (SS-OCT), have significantly improved the image resolution, reaching up to 3–5 µm [3,4]. Deeper ocular penetration with higher wavelength SS-OCT allows clinicians to visualize additional details of the choroid, i.e., the choriocapillaris, Haller’s layer, Sattler’s layer, choroidoscleral interface and even the scleral tissue in special scenarios [5]. Features like eye tracking and scanning the same area during follow up help the clinicians to accurately detect the subtle change at the site of pathology.

En-face OCT scans, also referred as C-scans, based on the coronal plane are generated post 3-D scan acquisition and are different compared to the routinely performed cross-sectional scans [6]. Another significant milestone deserving special mention is OCT angiography, which uses motion contrast to identify the blood flow in capillaries and has found wide usage to perform qualitative analyses on microaneurysms, macular edema, macular ischemia, retinal neovascularization and choroidal neovascular membranes, and quantitative analyses on the capillary density and measurement of chorioretinal lesion size [7]. Moreover, volumetric analysis with segmentation to specific depths provides significant advantages compared to dye-based angiography.

Initial OCT protocols were limited to the macular area covering an area of 6 × 6 mm. Subsequent improvements, especially wide-field OCT, provided additional insights on the peripheral retina, with clinical utility in eyes with peripheral retinal ischemia, retinal degeneration and peripheral choroidal lesions [8]. This was made possible with a much higher A-scan acquisition rate (>100,000/s) compared to earlier generation TD-OCT (approximately 400/s), thereby reducing scan acquisition time and increasing the field of view [8]. Now, multiple 12 × 12 mm or even 18- or 20-mm scans can be captured and montaged using additional software to create a much wider field of view reaching up to the equator and beyond. Another breakthrough is the integration of OCT imaging with surgical microscopes, which can be helpful in intraoperative anatomical assessment, especially in macular surgeries, for instance, on the macular hole and epiretinal membrane [9]. Surgeons can therefore assess the anatomical details intraoperatively and predict the surgical success rates. Though hand-held OCT and home-based OCT are other additions to the armamentarium, image resolution is typically lower than standard OCT machines [10]. Apart from retina and uveitis clinics, OCT is commonly used in glaucoma clinics to quantitively analyze retinal nerve fiber layer thickness and cornea clinics to assess the corneal thickness and anterior chamber depth.

Despite the innumerable benefits, high purchase and maintenance cost of OCT systems prevent widespread adoption in poor resource settings and low-income countries. Ongoing technical improvements can hopefully bring OCT size and cost down to more affordable levels. Moreover, the commercial instruments are bulky, not portable and tabletop mounted, which becomes challenging with pediatric patients, mentally disabled patients with the inability to fixate and elderly bedridden patients [10].

To conclude, OCT imaging, in a span of three decades, has undergone several modifications and now is a standard of care in ophthalmology clinics throughout the world. In this special edition, we focus on these recent advances in OCT technology.
